# A Celiac Cellular Phenotype, with Altered LPP Sub-Cellular Distribution, Is Inducible in Controls by the Toxic Gliadin Peptide P31-43

**DOI:** 10.1371/journal.pone.0079763

**Published:** 2013-11-22

**Authors:** Merlin Nanayakkara, Roberta Kosova, Giuliana Lania, Marco Sarno, Alessandra Gaito, Martina Galatola, Luigi Greco, Marialaura Cuomo, Riccardo Troncone, Salvatore Auricchio, Renata Auricchio, Maria Vittoria Barone

**Affiliations:** 1 Department of Translational Medical Science, University of Naples Federico II, Naples, Italy; 2 European Laboratory for the Investigation of Food Induced Disease, (ELFID) University of Naples Federico II, Naples, Italy; Bascom Palmer Eye Institute, University of Miami School of Medicine, United States of America

## Abstract

Celiac disease (CD) is a frequent inflammatory intestinal disease, with a genetic background, caused by gliadin-containing food. Undigested gliadin peptides P31-43 and P57-68 induce innate and adaptive T cell-mediated immune responses, respectively. Alterations in the cell shape and actin cytoskeleton are present in celiac enterocytes, and gliadin peptides induce actin rearrangements in both the CD mucosa and cell lines. Cell shape is maintained by the actin cytoskeleton and focal adhesions, sites of membrane attachment to the extracellular matrix. The locus of the human Lipoma Preferred Partner (LPP) gene was identified as strongly associated with CD using genome-wide association studies (GWAS). The LPP protein plays an important role in focal adhesion architecture and acts as a transcription factor in the nucleus. In this study, we examined the hypothesis that a constitutive alteration of the cell shape and the cytoskeleton, involving LPP, occurs in a cell compartment far from the main inflammation site in CD fibroblasts from skin explants. We analyzed the cell shape, actin organization, focal adhesion number, focal adhesion proteins, LPP sub-cellular distribution and adhesion to fibronectin of fibroblasts obtained from CD patients on a Gluten-Free Diet (GFD) and controls, without and with treatment with A-gliadin peptide P31-43. We observed a “CD cellular phenotype” in these fibroblasts, characterized by an altered cell shape and actin organization, increased number of focal adhesions, and altered intracellular LPP protein distribution. The treatment of controls fibroblasts with gliadin peptide P31-43 mimics the CD cellular phenotype regarding the cell shape, adhesion capacity, focal adhesion number and LPP sub-cellular distribution, suggesting a close association between these alterations and CD pathogenesis.

## Introduction

Celiac disease (CD) is characterized by mucosal inflammation due to the Th1 T cell response to certain gliadin peptides (e.g., the 33-mer A-gliadin peptide) [1] and the innate immune response to other gliadin peptides (e.g., A-gliadin peptide P31-43) [2]–[3]. Recent data have shown that gliadin peptides, particularly peptide P31-43, act as growth factors for cell lines, such as CaCo-2, and induce the proliferation of celiac crypt enterocytes [4]–[6]. Because both the 33-mer peptide containing P57-68 [7]–[8] and the 25-mer peptide containing P31-43 (P31-55) [9] are resistant to hydrolysis by gastric, pancreatic and intestinal proteases, these peptides remain active in vivo in the intestine after gluten ingestion.

Alterations of the actin cytoskeleton of epithelial cells are also observed in the celiac mucosa [10], and gluten-dependent shorter enterocyte heights have been described in CD [11]. Tight junction proteins are also altered in active CD, leading to increased permeability [12]–[13].

Furthermore, gliadin and gliadin peptides, including P31-43, interfere with actin rearrangements in both the CD mucosa and cell lines. The in vivo treatment of the small intestine of celiac disease patients with gliadin peptides causes the rearrangement of the actin cytoskeleton in enterocytes [14]–[15]. This phenomenon is also detectable in Caco-2 cells, a cell line commonly used to study gliadin effects on metabolism, barrier functions and apoptosis [16]–[17]. The treatment of CaCo-2 cells with peptic-tryptic gliadin digest and the truncated toxic peptide P31–43 causes rearrangements of the actin cytoskeleton similar to those induced by epidermal growth factor (EGF); [4]. Quantitative microscopic analysis revealed that gliadin directly damages F-actin and tight junction proteins in Lovo cells [18]. The direct interaction of gliadin peptides with actin impairs protein trafficking in COS1 cells [19]. Moreover, gliadin peptides induce cytoskeleton remodeling with altered motility in dendritic cells [20].

CD has been strongly associated with HLA-DQ 2/8 isotypes, which are necessary but not sufficient to produce this autoimmune disease. A total of 39 non-HLA susceptibility loci have recently been identified through repeated GWAS and explain approximately 10–14% of the CD heritability; combined with the HLA locus, only 50% of the CD heritability is explained [21]. The strongest non-HLA association signal maps to a 70-kb linkage disequilibrium (LD) block in intron 2 of the LPP gene [22].

LPP protein localizes at focal adhesions, which are sites of membrane attachment to the extracellular matrix, in cell-cell contacts, and in the cytoplasm and nucleus [23]. LPP protein is a proline-rich protein [24] previously identified in fibroblasts [25] that plays an important role in focal adhesion architecture as a versatile scaffolding and adaptor protein and also acts as a transcription factor in the nucleus [23].

As a scaffold and adaptor protein, LPP harbors various binding sites, one of which is a VASP (vasodilator-stimulated phosphoprotein)-binding site, which increases actin polymerization, leading to cell protrusions. After binding to LPP, VASP is recruited to cell adhesion sites, thus directing changes in actin dynamics [23].

The aim of the study was to analyze the actin cytoskeleton, cell shape, focal adhesions and adhesion to the substrate of skin fibroblasts, from CD patients and controls along with the sub-cellular distribution of the LPP protein. Finally we studied the effects of gliadin peptide P31-43 on the shape and adhesion of controls and CD fibroblasts. Skin fibroblasts, a cell compartment far from the main inflammation site, allowed to investigate differences at base line and following gliadin peptides stimulation in CD patients and controls.

## Materials and Methods

### Cell culture and treatments

Fibroblasts were cultured from skin biopsies obtained from CD patients and controls. We obtained fibroblasts [26] from seven celiac patients on a gluten-free diet (age range 17–43 years), six HLA DQ2-negative healthy controls (age range 25–30 years) and 1 HLA DQ2-positive healthy control (age 54 year). The patients were on a gluten-free diet for at least 4 years and showed normal biopsies (Marsh T0), anti-tTg antibody serum levels ranging between 0 and 1.6 U/ml and negative anti-endomisium antibodies (EMA). The DQ2-positive control did not score differently from the other controls in any of the assays described herein.

The skin explants were immediately placed in Dulbecco's Modified Eagle's Medium (DMEM) (GIBCO, San Giuliano Milanese, Italy) supplemented with 20% fetal bovine serum (FBS) (GIBCO, San Giuliano Milanese, Italy), 100 units/ml penicillin-streptomycin (GIBCO, San Giuliano Milanese, Italy), and 1 mM glutamine (GIBCO, San Giuliano Milanese, Italy) and incubated for 24 hours. Subsequently, each skin explant was divided into 50 small fragments, plated on Petri dishes and incubated in the presence of 95% oxygen and 5% CO2 at 37°C to allow adhesion and the subsequent release of fibroblasts. Several days later, the fibroblasts began to emerge from the fragments. When the fibroblasts reached confluence, the cells were harvested with trypsin and immediately frozen. In all experiments, the fibroblasts were used between the 2nd and 4th passage.

Lipopolysaccharide (LPS)-free synthetic peptides (Inbios, Naples, Italy) (>95% purity, evaluated using matrix-assisted laser desorption/ionization time-of-flight mass spectrometry) were obtained through Ultrasart-D20 filtration (Sartorius AG, Gottingen, Germany). The levels of LPS in these peptides were below the detection threshold, i.e., <0.20 EU/mg, assessed using the QCL-1000 kit (Cambrex Corporation, NJ). The P31-43 (sequence LGQQQPFPPQQPY) was used at a concentration of 100 µg/ml [4], [5], [6].

### Phalloidin, PAX and FAK staining

The fibroblasts were cultured on coverslips for 2 days and subsequently washed with PBS, fixed with 3% paraformaldehyde (Sigma Aldrich Co., Milan, Italy) and permeabilized with 0.2% Triton (Sigma Aldrich Co., Milan, Italy). For actin staining, the cells were incubated as previously described [4]. Briefly, the coverslips were treated with phalloidin-Alexa-488 for 40 min at room temperature (RT) in the dark. The coverslips were subsequently mounted on glass slides and observed by confocal microscopy (LSM 510 Zeiss). A total of 40 to 50 cells were observed in each sample, and all images were generated using the same confocal microscope. The cell area was analyzed using AIS Zeiss software.

For paxillin and FAK staining, the coverslips were fixed and permeabilized as previously described. The coverslips were subsequently treated with primary antibodies (mouse anti-paxillin and mouse anti-FAK, BD Biosciences, Pharmingen, Milan, Italy) for 1 h and after PBS washing with secondary antibody (anti-mouse TRITC, Invitrogen, Milan, Italy) in a dark humid chamber for 45 min. The coverslips were subsequently mounted on glass slides and observed by confocal microscopy (LSM 510 Zeiss). A total of 10 cells were observed in each sample, and all images were generated using the same confocal microscope. The number of focal adhesions per cell was assessed using AIS Zeiss software. The micrographs were magnified to the same size in all figures shown (63× objective).

### Colocalization of paxillin and LPP

For paxillin and LPP co-staining, the coverslips were fixed and permeabilized as previously described and subsequently treated with primary antibodies (mouse anti-paxillin, BD Biosciences, Pharmingen, Milan, Italy and rabbit anti-LPP, (Abcam, S. Francisco, Ca, USA) for 1 h, and after washing with PBS, the slides were incubated with secondary antibodies (anti-mouse TRITC and anti-rabbit Alexa-488, Invitrogen, Milan, Italy) in a dark humid chamber for 45 min. The coverslips were subsequently mounted on glass slides. Samples were examined using a Zeiss LSM 510 laser scanning confocal microscope. We used Argon/2 (458, 477, 488, and 514 nm) and HeNe1 (543 nm) excitation lasers, which were switched on separately to reduce crosstalk between the two fluorochromes. The green and red emissions were separated using a dichroic splitter (FT 560) and filtered through a 515 to 540-nm band-pass filter for green and >610-nm long-pass filter for red emission. A threshold was applied to the images to exclude approximately 99% of the signal observed in the control images. The weighted co-localization coefficient represents the sum of intensity of co-localizing pixels in channels 1 and 2 compared with the overall sum of the pixel intensities above the threshold. This value could be 0 (no co-localization) or 1 (all pixels co-localize). The bright pixels contributed more signal than the faint pixels. The co-localization coefficient represents the weighted colocalization coefficients of Ch1 (red) with respect to Ch2 (green).

### Nuclear-cytosol protein separation

Near-confluent cells were incubated in a 100-mm dish containing 1 ml of homogenization buffer (10 mM Tris-HCl [pH 7.4], 1 mM EDTA, 1 mM PMSF, 1 mM VO_4_, 100× Aprotinin, and 50× LAP; all purchased from Sigma, Milan, Italy, except for LAP, which was purchased from Roche, Milan, Italy) and homogenized using a Teflon homogenizer. The nuclear fraction was separated through centrifugation at 1,000× g (post-nuclear supernatant and nuclear fraction), and the pellet was incubated in 250 ml of homogenization buffer containing 0.4 M NaCl for 1 h on ice to disrupt the nuclei. After centrifugation at 105,000× g for 1 h, the supernatant (the crude nuclear extract) was recovered. The post-nuclear supernatant was centrifuged at 105,000× g for 1 h, and the cytosolic fraction (supernatant) was separated. The cytosolic and the crude nuclear extract were analyzed using immunoblotting.

### Western Blot

Briefly, the fibroblasts were cultivated in medium alone or in the presence of P31-43, as required. The cells were washed twice and resuspended in lysis buffer (Tris-HCl, pH 7.4, 50 mM, EDTA 1 mM, EGTA 1 mM, 5 mM MgCl_2_, 150 mM NaCl, 1% Triton, 1 mM PMSF, 1 mM VO_4_, 100× Aprotinin, and 50× LAP, all purchased from Sigma, Milan, Italy, except LAP, which was obtained from Roche, Milan, Italy). The cell lysates were analyzed with SDS-PAGE using running buffer (25 mM Trizma, 192 mM Glycine, and 0.1% SDS) and transferred onto nitrocellulose membranes (Whatman Gmbh, Dassel, Germany) using transfer buffer (25 mM Trizma, 192 mM Glycine, 0.1% SDS, and 20% methanol, all purchased from Sigma-Aldrich, Milan, Italy). The membranes were subsequently blocked using 5% non-fat dry milk solution and probed with anti-paxillin, anti-FAK, anti-pY-paxillin (BD Biosciences, Pharmingen, Milan, Italy), anti-pY-FAK (pY397) (BD Biosciences, Pharmingen, Milan, Italy) anti-tubulin (Sigma, Milan, Italy) anti-lamin A/C (Santa Cruz, Milan, Italy), or anti-LPP in blotting buffer (3% milk in TTBS, Tris-HCl, pH 8, 20 mM, NaCl 150 mM, and 0.1% Triton). All reagents were purchased from Sigma-Aldrich, Milan, Italy, except for SDS, milk, 2-mercaptoethanol and Triton, which were purchased from BioRad. The bands were visualized using ECL (GE Healthcare, Amersham, Buckinghamshire, UK) and exposure times of 2–10 min. The band intensity was evaluated by integrating all of the pixels of the band without the background to calculate the average of the pixels surrounding the band [4]–[6], [27]–[29].

### Adhesion assay

The 96-well plates (Nunc, Maxisorp, VWR International, Milan, Italy) were pre-coated overnight at 4°C with 0.5, 1.5, 10 and 50 µg/ml fibronectin in sterile water. The well plates were washed twice with sterile PBS and blocked for 1 h at RT with 5% BSA in PBS. All the experiments were performed in duplicate. After blocking, the well plates were washed twice with SFR (solution for resuspension, 5 mg/ml BSA, 5 mM glucose, and 0.3 mM MgCl_2_) and 10,000 cells in 100 µl SFR per well were added. The cells were incubated for 1 h at room temperature. After incubation, the cells were washed twice in PBS, and 100 µl of crystal violet solution containing 79.5% PBS, 20% methanol and 0.5% crystal violet (Sigma, Milan, Italy) was added and incubated overnight at room temperature. The next day, the plates were washed vigorously with PBS, followed by a final wash with sterile water. The number of cells adhering to fibronectin was determined by counting the number of adherent cells in 5 random photos obtained from 2 similar 96-well plates at 40× magnification under each test condition. Five random photos were counted from at least 3 independent experiments for each sample, and the mean and standard deviation were calculated [30].

### Gene expression studies

Total RNA was extracted from fibroblasts from GFD CD patients and controls using TRIZOL Reagent (Ambion®-Life Technologies). The quantity of RNA was measured using a Nanodrop® spectrophotometer, and subsequently, the RNA quality was analyzed using agarose gel electrophoresis in Tris/Borate/EDTA buffer (TBE). The RNA (1 µg) was reverse transcribed into cDNA using the High Capacity cDNA Reverse Transcription kit according to the manufacturer's protocol. The experiments were performed with a 7900HT Fast Real-Time PCR system using the TaqMan® Gene Expression Assay and approximately 40 ng of cDNA according to the manufacturer's protocol. The gene expression assay used for LPP gene is Hs00944352_m1 (Life Technologies), the probe is located in the 3′ region, spanning exons 8 and 9. The relative expression was calculated using the comparative Ct method. The expression of each gene was normalized to an endogenous housekeeping gene (*GUSb*). The relative quantification was performed using the ΔΔCt method. The SDS software (ABI, version 1.4 or 2.4) was used to analyze the raw data, and subsequently, an additional statistical analysis was performed on GraphPad Prism 5.01®.

### LPP Genotyping

The fibroblasts samples from 7 patients and 5 controls were genotyped for the LPP SNP rs1464510, which is associated with CD [31]. Genomic DNA was extracted from fibroblasts using the Genomic DNA Extraction kit (QIAamp DNA Mini Kit; Qiagen, Hilden, Germany) according to the manufacturer's instructions. The quantity of DNA extracted was estimated using a Nanodrop® spectrophotometer. The genotyping reactions were performed with 50 ng of genomic DNA using real-time PCR allelic discrimination TaqMan genotyping assays (Applied Biosystems, Foster city, USA) and a 7900HT Fast Real-Time PCR System (Applied Biosystems) in a final volume of 15 µL according to the manufacturer's instructions. The results were analyzed using the SDS software ver. 2.3.

### Ethics statement

All adult subjects provided written informed consent to use the biopsies in this study. Parents or tutors provided written informed consent for subjects under 18 years of age. The protocol for this study was approved by the Ethical Committee of the University “Federico II”, Naples, Italy (ethical approval: C.E. n. 230/05).

### Statistical analyses

GraphPad Prism 5.lnk (GraphPad Software, San Diego, CA, USA) was used for statistical analysis and graphical representation. The statistical analysis of the differences was performed using Student's *t*-test. A *p* value<0.05 was considered statistically significant.

## Results

### Cell shape and area of fibroblasts from CD patients were altered compared to the controls

**Figure 1:**
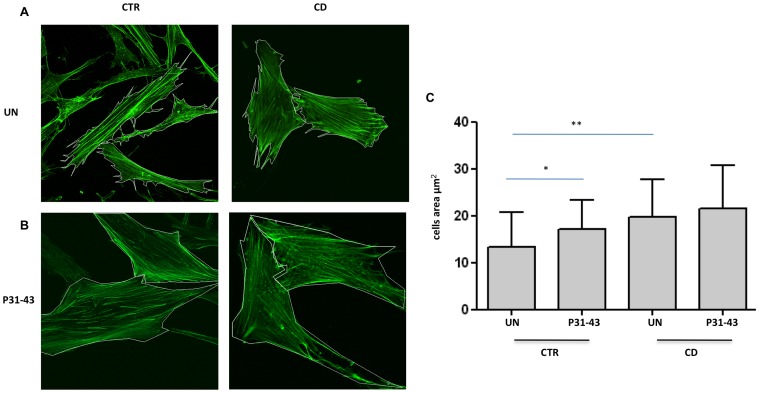
Cell shape and area of fibroblasts from CD patients and controls before and after P31-43 treatment. A. Cell shape and area are altered in fibroblasts from CD patients with respect to controls (CTR). Confocal immunofluorescence images of fibroblasts from CD patients and controls stained with Phalloidin-FITC to highlight F-actin. White lines identify the cell area. Representative fields. B. Treatment with P31-43 alters the shape and area of control fibroblasts. Confocal immunofluorescence images of fibroblasts from CD patients and controls treated with P31-43 for 30 min and stained with Phalloidin-FITC to highlight F-actin. White lines identify the cell area. Representative fields. C. Statistical analysis of 30 fibroblasts from several fields of 3 independent experiments from 6 patients and 6 controls. The area of the cells was analyzed using LSM-Zeiss confocal software. Columns represent the means, and the bars represent the standard deviation of the fibroblasts area. The area of the cells was analyzed using LSM-Zeiss confocal software. Columns represent the means, and the bars represent the standard deviation. Student's *t*-test. * = *p*<0.05; ** = *p*<0.01.

The cell shape and actin rearrangements in fibroblasts were analyzed through phalloidin-FITC staining. As shown in figure 1A, the CD cells had shortened actin stress fibers and appeared less elongated that the controls. The analysis of the cell area (figure 1C) revealed that CD fibroblasts showed a statistically significant increase with respect to the controls (mean and SD of the area: controls 13.3+/−7.35 mm^2^; CD patients 19.7+/−6.24 mm^2^).

### Treatment with gliadin peptide P31-43 altered the cell shape and area of the control fibroblasts

The cell shape and actin rearrangements of fibroblasts were analyzed using phalloidin-FITC staining after treatment with P31-43 for 30′. As shown in figures 1 B and C, the shape and area of the CD fibroblasts did not substantially change after P31-43 treatment (21.5+/−9 mm^2^). Instead, control fibroblasts treated with P31-43 exhibited shortened actin stress fibers and appeared less elongated than the untreated fibroblasts. The analysis of the cell area (figure 1C) revealed that the control fibroblasts exhibited a statistically significant increase in area after P31-43 treatment compared with untreated control fibroblasts, almost reaching the CD fibroblast area (mean and SD of the area of P31-43-treated controls: 17.1+/−7.94 mm^2^).

### The focal adhesion number was increased in fibroblasts from CD

**Figure 2:**
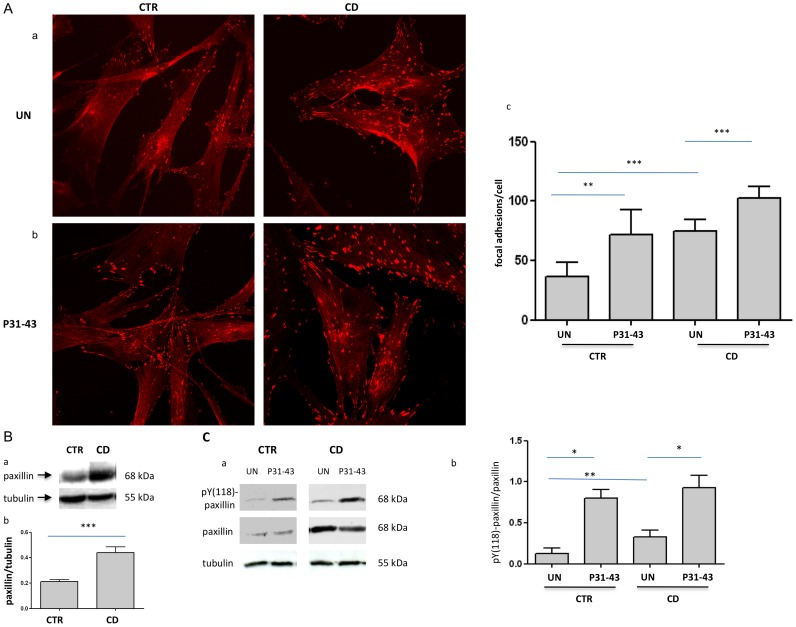
Analysis of the focal adhesion number and paxillin protein levels in CD and controls fibroblasts before and after P31-43 treatment. A. Increase of the focal adhesion number in CD, as revealed through paxillin staining before and after P31-43 treatment. a) Confocal immunofluorescence images of fibroblasts from CD patients and controls stained with antibodies against paxillin. Representative fields. b) Confocal immunofluorescence images of fibroblasts stained with antibodies against paxillin from CD patients and controls treated with P31-43 for 30 min. Representative fields. c) Statistical analysis of the number of paxillin-positive focal adhesions per cell before and after P31-43 treatment. Focal adhesions of 30 fibroblasts of several fields from 3 independent experiments from 6 patients and 6 controls were counted. Columns represent the means, and the bars represent the standard deviations. Student's *t*-test. ** = *p*<0.01; *** = *p*<0.001. B. Paxillin protein levels are increased in fibroblasts from CD patients with respect to controls. a) Western blot analysis of fibroblast protein lysates from controls and CD patients. The upper line is stained for paxillin, and the lower line is stained for tubulin. Representative experiment. b) Densitometric analysis of western blots stained for paxillin in fibroblasts from 6 CD patients and 6 controls. Paxillin levels were normalized to the tubulin levels. Columns represent the means, and the bars represent the standard deviation. Student's *t*-test. *** = *p*<0.001. C. Paxillin phopshorylation is increased in CD fibroblasts and is further increased after P31-43 treatment for 30′. a) Western blot analysis of fibroblast protein lysates from controls and CD patients. The upper line is stained for pY-paxillin, the middle line is stained for paxillin, and the lower line is stained for tubulin. Representative experiment. b) Densitometric analysis of western blots stained for pY-paxillin and paxillin in fibroblasts from 5 CD patients and 5 controls. pY-paxillin levels were normalized to the paxillin levels. Columns represent the means, and the bars represent the standard deviation. Student's *t*-test. * = *p*<0.05; ** = *p*<0.01.

**Figure 3:**
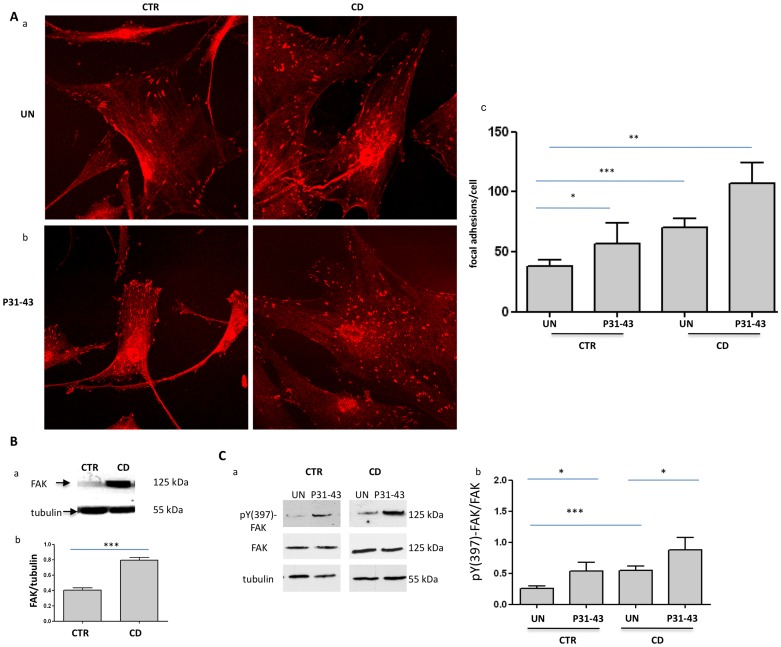
Analysis of the focal adhesion number and FAK levels in CD and controls fibroblasts before and after P31-43 treatment. A. Increase of focal adhesion number in CD, as revealed through FAK staining before and after P31-43 treatment. a) Confocal immunofluorescence images of fibroblasts from CD patients and controls stained with antibodies against FAK. Representative fields. b) Confocal immunofluorescence images of fibroblasts stained with antibodies against FAK from CD patients and controls treated with P31-43 for 30 min. Representative fields. c) Statistical analysis of the number of FAK-positive focal adhesions per cell before and after P31-43 treatment. Focal adhesions of 30 fibroblasts of several fields from 3 independent experiments with 6 patients and 6 controls were counted. Columns represent the means, and the bars represent the standard deviations. Student's *t*-test. * = *p*<0.05; ** = *p*<0.01; *** = *p*<0.001. B. FAK protein levels are increased in fibroblasts from CD patients with respect to the controls. a) Western blot analysis of fibroblast protein lysates from controls and CD patients. The upper line is stained for FAK, and the lower line is stained for tubulin. Representative experiment. b) Densitometric analysis of western blots stained for FAK in fibroblasts from 5 CD patients and 5 controls. FAK levels were normalized to the tubulin levels. Columns represent the means, and the bars represent the standard deviations. Student's *t*-test. *** = *p*<0.001. C. FAK phopshorylation is increased in CD fibroblasts and is further increased after P31-43 treatment for 30′. a) Western blot analysis of fibroblasts proteins lysates from controls and CD patients treated or not with P31-43. The upper line is stained for pY-FAK, the middle line is stained for FAK and the lower line is stained for tubulin. Representative experiment. b) Densitometric analysis of western blots stained for pY-FAK and FAK in fibroblasts from 5 CD patients and 5 controls. pY-FAK levels were normalized to the FAK levels. Columns represent the means, and the bars represent the standard deviation. Student's *t*-test. * = *p*<0.05; *** = *p*<0.001.

The cell shape is maintained through the actin cytoskeleton and adhesion to the substrate [32]. Thus, we analyzed the expression of paxillin and FAK, two focal adhesion markers, to determine the focal adhesion of CD fibroblasts with respect to control cells [33]. Figures 2 and 3 present the results of the immunofluorescence analysis of focal adhesions stained with antibodies against paxillin and FAK. Both paxillin- (figure 2Aa) and FAK (figure 3Aa)-positive focal adhesions were significantly increased in CD fibroblasts with respect to the controls (figure 2, 3 Ac). Paxillin-positive focal adhesions were 36.7+/−11.59 per cell in control fibroblasts and 75+/−9.41 in CD fibroblasts (figure 2 Ac). Similarly, FAK-positive focal adhesions were 37.7+/−5.18 in control fibroblasts and 70+/−7.39 in CD fibroblasts (figure 3 Ac). These differences were statistically significant. The number of focal adhesions was also increased when expressed per 100 µm^2^. Indeed, the paxillin-positive focal adhesions were 2.6+/−0.7 per 100 µm^2^ in control fibroblasts and 5.3+/−0.6 per 100 µm^2^ in CD fibroblasts. Similarly, FAK-positive focal adhesions were 2+/−0.3 per 100 µm^2^ in control fibroblasts and 5.8+/−0.5 per 100 µm^2^ in CD fibroblasts. These differences were also statistically significant. Co-staining of FAK and actin further confirm increased number of focal adhesion in CD fibroblasts and shortened actin stress fibres (Figure S1).

Western blot analysis revealed a two-fold increase of paxillin (figure 2B) and FAK protein (figure 3B) in fibroblasts from CD patients compared with controls, and an increase in the phosphorylated forms of these proteins was also observed (figure 2, 3 C).

### Treatment with gliadin peptide P31-43 increased both paxillin- and FAK-positive focal adhesions in CD and control fibroblasts

Figures 2Ab and 3Ab show the results of the immunofluorescence analysis of focal adhesions stained with antibodies against paxillin and FAK after treatment with P31-43 for 30 min. Treatment with P31-43 increased both paxillin- (figure 2Ac) and FAK (figure 3Ac)-positive focal adhesions in CD fibroblasts and controls.

After treating control fibroblasts with P31-43, paxillin-positive focal adhesions were 71.6+/−21.17 per cell (figure 2Ac). Similarly, FAK-positive focal adhesions were 56.5+/−17.68 per cell (figure 3Ac).

In CD fibroblasts, P31-43 treatment increased paxillin-positive focal adhesions to 102.5+/−9.9 per cell (figure 2Ac). Similarly, FAK-positive focal adhesions increased to 107+/−17.1 per cell after P31-43 treatment. Moreover, treatment with P31-43 for 30 min significantly increased the phosphorylation of both paxillin and FAK (figure 2 and 3C).

### Subcellular localization of LPP in CD fibroblasts with respect to controls

**Figure 4:**
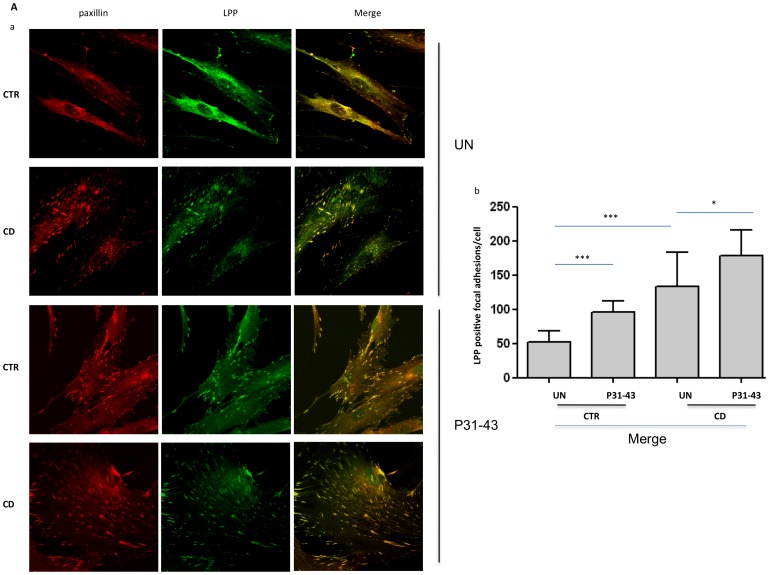
Analysis of LPP levels and sub-cellular distribution in CD and controls fibroblasts before and after P31-43 treatment. A. Increased focal adhesion localization of LPP in CD fibroblasts with respect to controls, as revealed through paxillin co-staining. a) Confocal immunofluorescence images of fibroblasts from CD patients and controls treated or not with P31-43 for 30 min and stained with antibodies against paxillin (red) and LPP (green); the merge of the red and green fields is shown in yellow. Representative fields. b) Statistical analysis of the number of LPP/paxillin-merged positive focal adhesions per cell. Focal adhesions of 30 fibroblasts from several fields from 3 independent experiments with 5 patients and 5 controls were counted. Columns represent the means, and the bars represent the standard deviations. Student's *t*-test. * = *p*<0.05;*** = *p*<0.001. B. LPP protein levels were not increased in the fibroblasts from CD patients with respect to the controls and did not vary after 24 h treatment with P31-43. a) Western blot analysis of fibroblasts protein lysates from controls and CD patients treated or not with P31-43. The upper line was stained for LPP, and the lower line was stained for tubulin. Representative experiment. b) Densitometric analysis of western blots stained for LPP in fibroblasts from 5 CD patients and 5 controls treated or not with P31-43. LPP levels were normalized to tubulin levels. Columns represent the means, and the bars represent the standard deviations. Student's *t*-test. C. Decreased localization of LPP in the nuclear fraction of CD fibroblasts. a) Western blot analysis of LPP after separating the nuclear and cytosolic protein fractions. The upper lines were stained for LPP, and the lower lines were stained for tubulin and lamin A/C, which were used as loading controls for the cytosol and nuclear fractions, respectively. Representative experiment. b) Densitometric analysis of western blots stained for LPP in fibroblasts from 5 CD patients and 5 controls. The LPP levels were normalized in each protein fractions to the loading controls. Columns represent the means, and the bars represent the standard deviation. Student's *t*-test. * = p<0.05; ** = *p*<0.01.

The LPP gene is strongly associated with CD [22], its protein is localized at focal adhesions and transiently in the nucleus, integrating signals from the extracellular matrix through the cell surface into the nucleus [23]. Therefore, we analyzed the localization of LPP in focal adhesions and determined the expression and sub-cellular distribution of this protein in CD fibroblasts and controls (figure 4). The immunofluorescence analysis of focal adhesions stained with an antibody against paxillin and LPP revealed that LPP co-localized with paxillin in focal adhesions. The weighted co-localization coefficient was 0.75 for the controls and 0.85 for the CD fibroblasts (figure 4Aa).

Moreover, a statistically significant increase in the number of focal adhesions co-stained with LPP in CD fibroblasts was observed (figure 4Ab). In fact, the number of focal adhesions per cell, co-stained for paxillin and LPP, was 51.5+/−16.1 in controls and 133+/−50.7 in CD fibroblasts; this difference was statistically significant. The expression of the two markers of focal adhesions, paxillin and FAK, was increased in CD fibroblasts; therefore, we analyzed LPP protein and mRNA levels. The mRNA expression of LPP was increased in CD patients compared with controls, although this difference was not statistically significant (figure S2A).

No increase in LPP protein expression was observed in CD patients compared to controls (figure 4B), suggesting that the compartmentalization of this protein in focal adhesions in CD patients reflected differences in the sub-cellular distribution of LPP.

We also analyzed the sub-cellular distribution of LPP protein by separating the nuclear and cytosolic protein fractions from CD fibroblasts and controls. Figure 4C shows the intracellular distribution of LPP protein in the different protein fractions. LPP protein was significantly reduced in the nuclear fraction of CD fibroblasts compared with controls. A corresponding increase in LPP protein was observed in the cytosolic fraction, where the focal adhesion proteins are expected to be found. Taken together, these data indicate that the sub-cellular distribution of LPP is altered in CD, with increased LPP in focal adhesions and reduced LPP in the nucleus.

### Treatment with P31-43 influenced the sub-cellular localization of LPP in CD fibroblasts and controls

We subsequently analyzed the effect of treatment with the gliadin peptide P31-43 on LPP localization at the level of the focal contacts. Figure 4Aa shows the co-localization of LPP with paxillin after P31-43 treatment. A statistically significant increase in the number of focal adhesions co-stained with LPP was observed after P31-43 treatment in fibroblasts from both controls and CD patients (figure 4Ab) (95.8+/−16.6 for the controls and 178+/−37.3 for the CD patients), suggesting that P31-43 treatment reproduced the CD phenotype in controls, with increased LPP localization in focal adhesions. The weighted co-localization coefficient was 0.75 for the controls and 0.87 for the CD fibroblasts.

LPP protein was not increased after 30-min (not shown) or 24-h (figure 4B) P31-43 treatment.

### Adhesion on fibronectin was altered in CD patients

**Figure 5:**
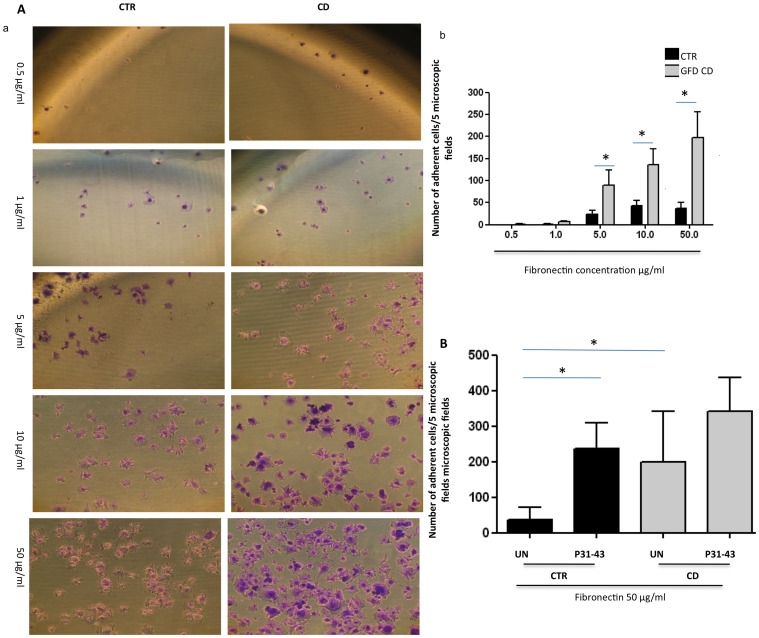
Fibronectin adhesion of CD fibroblasts respect to controls. A. CD fibroblasts adhered more than controls to fibronectin. a) Transmitted light images of crystal violet stained fibroblasts from CD patients and controls, seeded for 1 h on increasing concentrations of fibronectin, as indicated. Representative fields. b) Statistical analysis of the number of adherent fibroblasts from CD patients and controls seeded on different concentrations of fibronectin, as indicated. For each fibronectin concentration, the fibroblasts of 5 fields from 3 independent experiments with 5 patients and 5 controls were counted. Columns represent the means, and the bars represent the standard deviations. Student's *t*-test. * = *p*<0.01. B. P31-43 increased adhesion to fibronectin. Statistical analysis of the number of adherent fibroblasts from CD patients and controls seeded on 50 µg of fibronectin after P31-43 treatment for 1 h. Fibroblasts in 5 fields from 3 independent experiments with 5 patients and 5 controls were counted. Columns represent the means, and the bars represent the standard deviations. Student's *t*-test.

Focal adhesion and actin remodeling control cell adhesion and motility [33]. Therefore, we examined whether the increased number of focal adhesions affected the adhesion (figure 5A) of fibroblasts from CD patients. Adhesion assays applying different concentrations of fibronectin (from 0.5 to 50 µg/ml) for 1 h were used to examine the adhesion of fibroblasts from patients and controls. The number of adherent fibroblasts in 5 photographic fields was increased in CD in a concentration-dependent manner from 5 to 50 µg/ml of fibronectin compared with the controls. At the maximum concentration of 50 µg/ml of fibronectin, 198+/−143 adherent cells in 5 microscopic fields were counted in CD patients compared with 37+/−34 fibroblasts in 5 microscopic fields for the controls (figure 5Ab).

### P31-43 treatment increased adhesion on fibronectin

An adhesion assay using 50 µg/ml of fibronectin for 1 h showed the adhesion ability of fibroblasts from patients and controls after P31-43 treatment. The number of adherent fibroblasts in 5 photographic fields increased after P31-43 treatment. The adhesion of the control fibroblasts increased from 37+/−34 to 235+/−74 cells per 5 microscopic fields; the difference was statistically significant. Interestingly, the number of control fibroblasts adhering on fibronectin after P31-43 treatment was similar to that of the untreated CD fibroblasts. Treatment with P31-43 further increased CD fibroblast adhesion on fibronectin to 341+/−95 cells per 5 microscopic fields, although this increase was not statistically significant (figure 5B).

### LPP genotype in CD and controls fibroblasts

The LPP-SNP (Single Nucleotide Polymorphism) rs1464510, the “A” risk allele, has been associated with an enhanced risk for CD disease [34]. Therefore, we genotyped both CD and control fibroblasts to examine the correlation between the LPP “A” risk allele and the mRNA levels (figure S 2B). One control and 3 CD patients exhibited the “AA” genotype, but none of these individuals showed any clear difference in the mRNA levels. No differences were observed between the three AA patients and the other CD patients with respect to cell area, focal adhesion number, fibronectin adhesion, LPP protein levels and sub-cellular distribution (data not shown).

## Discussion

In this study, we described a “CD cellular phenotype” characterized by altered cell shape and actin distribution, increased adhesion structures and proteins, including LPP, and increased adhesion. The gliadin peptide P31-43 induces the same phenotype in control fibroblasts.

Alterations in the actin cytoskeleton and tight junctions have previously been described in CD intestinal mucosa [4], [10], [15], indicating that cytoskeletal rearrangements and cell contacts might be involved in celiac lesions. We observed an altered cell shape with shorter actin stress fibers and increased cell area in CD fibroblasts, demonstrating that alterations in the cell structure can be observed in cells far from the intestinal lesion and in the absence of gliadin peptides. We observed similar results in a different cell population: CD dendritic cells (DCs) showed a constitutive alteration of the cytoskeleton and the cell shape after adhesion to fibronectin. When in contact with fibronectin, the DCs from CD patients (both active and on a gluten-free diet) were more elongated with long dendrites compared with controls (unpublished data).

Cell structure is maintained through the actin cytoskeleton and focal contacts sites with the extracellular environment [32]. These specialized cell adhesion sites play a role in the architectural organization and polarity of the cells.

Focal adhesion kinase (FAK) is a marker for focal contacts sites, and FAK-Src signals regulate cell motility and focal contact localization. At the point of contact with the substrate, integrin clustering promotes FAK auto phosphorylation at Tyr-397, facilitating the formation of the focal adhesions at substrate contact points and actin filament initiation sites. Paxillin is another protein that acts at focal contact sites, and this protein binds to FAK and integrin [33].

Paxillin- and FAK-positive focal adhesions were increased in CD fibroblasts when expressed per cell or per unit of surface area. We also observed an increase of the FAK and paxillin protein levels and of the phosphorylation of these proteins in CD fibroblasts, suggesting an over representation of these specialized cell adhesion sites in the absence of gluten and at locations far from the main inflammation site.

Alterations of the actin cytoskeleton and the focal adhesions observed in CD fibroblasts prompted an examination of the expression and distribution of LPP, which is also localized at the contact sites of these cells. LPP plays a role in both the function of cell adhesion sites and the genetics of CD.

Specialized cell adhesion sites are dynamic units that are directly involved in communicational processes. Adhesion receptors and their cytoskeletal partners regulate the nucleo-cytoplasmic trafficking of signaling proteins and therefore influence gene expression [23]. LPP possesses a nuclear export signal and shuttle from nucleus to focal adhesions. Within the nucleus, LPP acts as a transcription factor, enhancing the transcriptional activity of several genes, alone or in cooperation with other proteins [23]. LPP also plays an important role in tumor metastasis and human epidermal growth factor receptor 2/neu-mediated mammary oncogenesis [35]. Recently, LPP was identified as a substrate of the protein-tyrosine-phosphatase 1B, a negative regulator of multiple signaling pathways downstream of receptor tyrosine kinases, and functionally associated with Ras signaling [36]–[37].

The locus of the human LPP gene was strongly associated with CD through GWAS studies in different populations [21]. An association between LPP-SNP rs1464510 “AA” and CD has also been revealed in potential CD (patients with normal intestines and HLA DQ2/8 and anti-tTg antibodies in the serum) [38]. None of the CD-associated SNPs mapped to the coding sequence of LPP. More recently four SNPs were associated with transcription factor-binding sites, suggesting that the dysregulation of transcription-binding properties might be the causal mechanism underlying the association of CD with the LPP region [39]. Therefore, we studied the localization of LPP in focal adhesions and the expression and sub-cellular localization of this protein in CD fibroblasts compared with controls. We found that LPP co-localized with paxillin in focal adhesions and that the number of paxillin focal adhesions co-stained with LPP was increased in CD fibroblasts. Although paxillin and FAK proteins were increased, there was no increase of LPP protein and mRNA levels in CD fibroblasts, indicating a different sub-cellular distribution of LPP in CD cells with respect to the controls.

The different distribution of LPP protein in CD cells might have several biological effects. For example, the increased LPP localization at the focal adhesions could alter cellular adhesion, motility and shape, and the reduced amount of LPP in the nucleus could alter the transcriptional activity of this protein. We found that the levels of LPP protein, in the nuclear fraction, was reduced in CD fibroblasts compared with control cells. This result was consistent with the hypothesis that the altered transcriptional activity of LPP might be involved in celiac disease [23]. No differences between patients with the LPP “AA” risk allele and other CD patients were observed with regard to the sub-cellular distribution of LPP protein.

Very likely alterations of phospho-paxillin and phospho-FAK and delocalization of LPP are the consequences of complex signaling involving several pathways including small-GTP binging proteins, known regulators of these cell activities. Moreover Rho A and B have been described to be involved in CD patients transglutaminase-2 targeted autoantibodies effects on endothelial differentiation [40]–[41].

In the present study, we described a “CD cellular phenotype” involving LPP protein, whose gene is strongly associated with CD. This phenotype is present in all CD subjects, independent of the at-risk genotype (AA). Similarly, in other polygenic intestinal inflammatory diseases, such as Crohn's disease, a disease phenotype present in all patients has been observed [42]. Indeed, an alteration of the autophagy of the Paneth cells is present in Crohn's biopsies, independent of the genetic variants of the autophagic pathway observed in Crohn's patients. This observation indicates that a disease cellular phenotype could be present in all subjects with polygenic diseases. These results suggest that SNPs could serve as a marker for the metabolic pathways involved in the disease, although their association was only observed in a small percentage of the patients.

Focal contact sites are located at the point of contact of the cells with the extracellular environment. Therefore, we evaluated the adhesion of CD and control fibroblasts to fibronectin and found that a larger number of CD fibroblasts adhere to the substrate compared to the controls.

We subsequently challenged both the controls and CD fibroblasts with gliadin peptide P31-43. Interestingly, the toxic peptide P31-43 induced the “cellular CD phenotype” in control fibroblasts. Indeed, treatment with gliadin peptide P31-43 altered the cell shape, with shortened actin stress fibers and increased area, in the control fibroblasts. In addition, the focal contact sites were also altered in controls fibroblasts after P31-43 treatment, with increased focal adhesion numbers and increased paxillin and FAK phosphorylation.

Interestingly, the distribution of LPP was affected after P31-43 treatment, with increased co-localization of LPP protein with paxillin at focal adhesions. Moreover, the adhesion of controls fibroblasts to fibronectin increased after treatment with P31-43.

Taken all together, these results suggest that the “toxic” gliadin peptide P31-43 is active in the same cellular pathways that involve cell shape and adhesion, which are constitutively altered in CD cells. Interestingly, P31-43 treatment altered the number of the focal adhesions, phosphorylation of paxillin and FAK, LPP distribution and adhesion to fibronectin also in CD fibroblasts.

Although the mechanism by which gliadin peptides affect cell shape and adhesion has not been elucidated, this might reflect the known effects of P31-43 on endocytic functions, actin remodeling, epidermal growth factor (EGF) signaling [4]–[6], [19], [43]–[46]. Moreover constitutive activation of the EGFR/ERK pathway in CD fibroblasts and enterocytes from CD biopsies has been demonstrated, independent of the stage of the disease, the gluten content of the diet and the site of inflammation [47].

Alterations of the cell shape and interactions with the extracellular matrix have recently been described by measuring collagen production and cellular displacement in fibroblasts from intestinal biopsies of CD patients with respect to controls [48]. The structural and functional changes of fibroblasts might play a role in the alterations of the proliferation-differentiation program of the enterocytes present in CD and the establishment of celiac intestinal lesions [49].

In conclusion, we described constitutive alterations involving LPP in the cell shape and adhesion of CD fibroblasts that occurred in all CD patients. This “CD cellular phenotype” is reproduced in control cells through treatment with P31-43, implying a close association between these alterations and CD pathogenesis.

## Supporting Information

Figure S1 Costaining of FAK and actin in CD and controls fibroblasts. Confocal immunofluorescence images of fibroblasts from CD patients and controls stained with antibodies against FAK to highlight focal adhesions and Phalloidin-FITC to highlight F-actin. Representative fields of 3 independent experiments from 6 patients and 6 controls.
(TIFF)

Figure S2 LPP genotyping assay and gene expression experiments in CD and controls fibroblasts. A: Quantitative PCR analysis of LPP mRNA in fibroblasts from CD patients and controls. RQ = relative quantity of LPP mRNA. The round symbols and the squared symbols represented the controls and the patients respectively. Different shades of gray represented the three genotypes of LPP as in figure B. Horizontal bars and vertical bars represented the mean and the standard deviation respectively. Differences between LPP levels in patients and controls were not statistically significant (p<0,01). Student *t*-test. B: Distribution of the three genotypes (AA, AC, CC) for the SNP of LPP in controls and CD subjects. In grayscale are depicted the different genotypes. The risk “A” alleles produced an enhanced risk and was significantly associated with CD *(Izzo V, Pinelli M, Tinto N, Esposito MV, Cola A, Sperandeo MP, Tucci F, Cocozza S, Greco L, Sacchetti L.* (*2011) Improving the estimation of celiac disease sibling risk by non-HLA genes. PLoS One. 6: e26920. doi: 10.1371)*
(TIFF)
