# Supplementary figures and images for: A Celiac Cellular Phenotype, with Altered LPP Sub-Cellular Distribution, Is Inducible in Controls by the Toxic Gliadin Peptide P31-43
Source: PLoS One. 2013 Nov 22;8(11):e79763. doi: 10.1371/journal.pone.0079763 (PMC3838353; doi:10.1371/journal.pone.0079763)

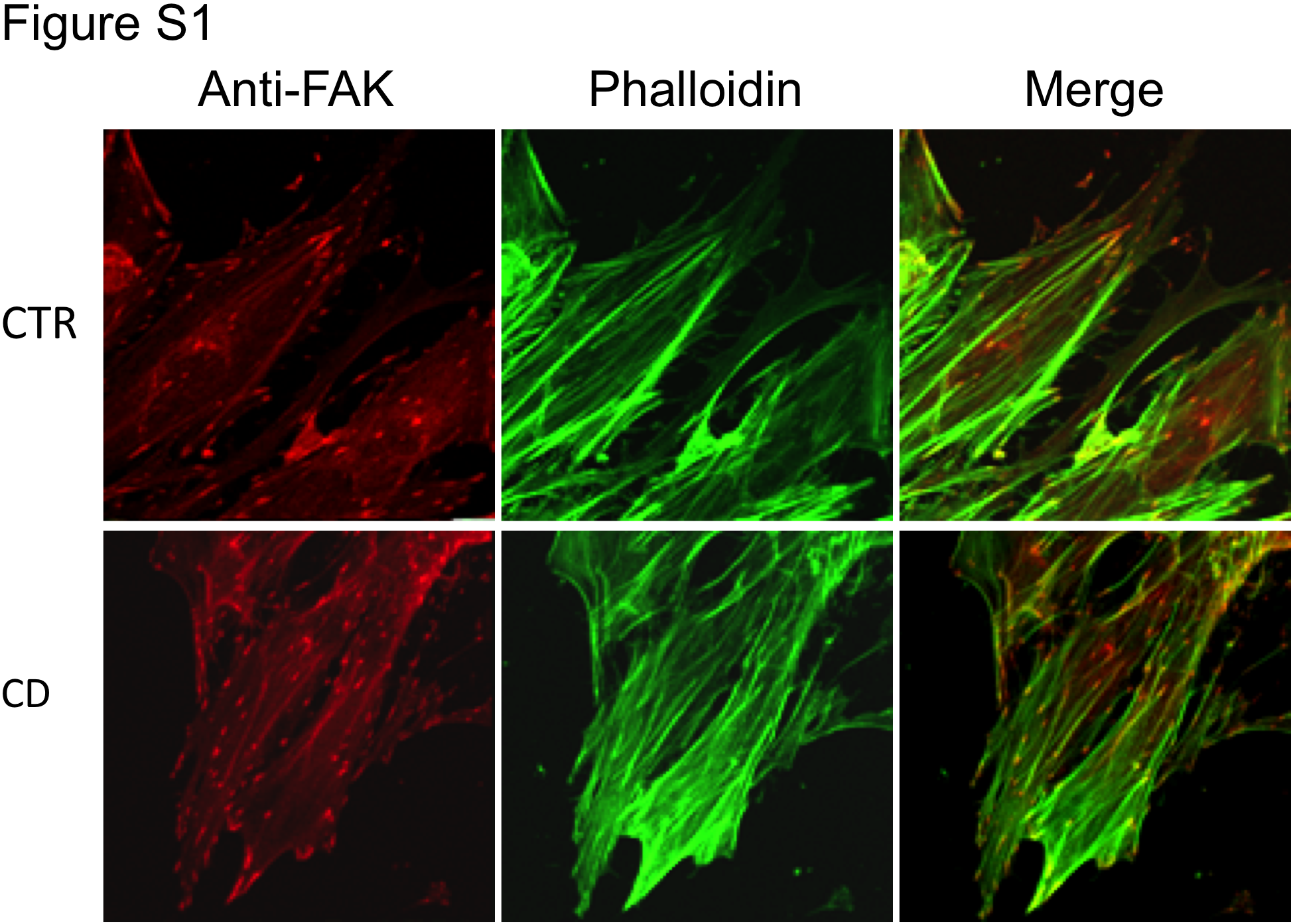

Supplement: Figure S1 — Costaining of FAK and actin in CD and controls fibroblasts. Confocal immunofluorescence images of fibroblasts from CD patients and controls stained with antibodies against FAK to highlight focal adhesions and Phalloidin-FITC to highlight F-actin. Representative fields of 3 independent experiments from 6 patients and 6 controls. (TIFF) [file pone.0079763.s001.tif]

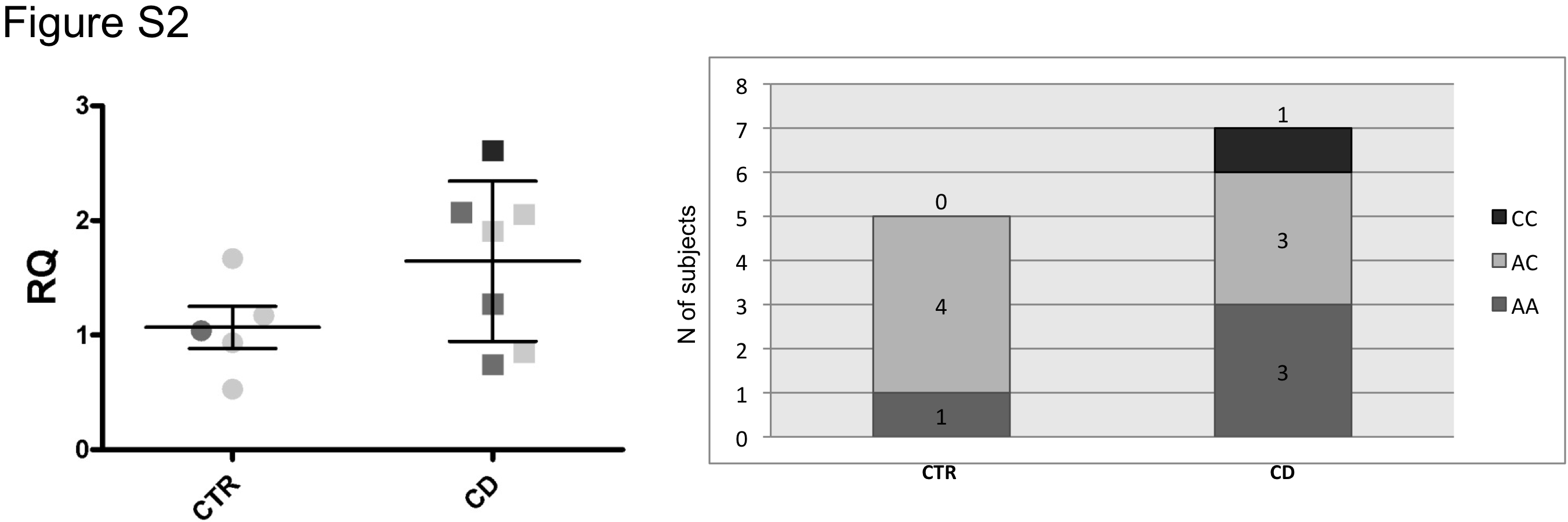

Supplement: Figure S2 — LPP genotyping assay and gene expression experiments in CD and controls fibroblasts. A: Quantitative PCR analysis of LPP mRNA in fibroblasts from CD patients and controls. RQ = relative quantity of LPP mRNA. The round symbols and the squared symbols represented the controls and the patients respectively. Different shades of gray represented the three genotypes of LPP as in figure B. Horizontal bars and vertical bars represented the mean and the standard deviation respectively. Differences between LPP levels in patients and controls were not statistically significant (p<0,01). Student t-test. B: Distribution of the three genotypes (AA, AC, CC) for the SNP of LPP in controls and CD subjects. In grayscale are depicted the different genotypes. The risk “A” alleles produced an enhanced risk and was significantly associated with CD (Izzo V, Pinelli M, Tinto N, Esposito MV, Cola A, Sperandeo MP, Tucci F, Cocozza S, Greco L, Sacchetti L. (2011) Improving the estimation of celiac disease sibling risk by non-HLA genes. PLoS One. 6: e26920. doi: 10.1371) (TIFF) [file pone.0079763.s002.tif]
